# Biomass-Derived Magnetic Fe_3_O_4_/Biochar Nanoparticles from Baobab Seeds for Sustainable Wastewater Dye Remediation

**DOI:** 10.3390/ijms26178499

**Published:** 2025-09-01

**Authors:** Samah Daffalla

**Affiliations:** Department of Environment and Agricultural Natural Resources, College of Agricultural and Food Sciences, King Faisal University, P.O. Box 400, Al-Ahsa 31982, Saudi Arabia; sbalal@kfu.edu.sa

**Keywords:** baobab seeds, magnetic biochar, nanoparticles, Congo red, oxidation processes, environmental remediation

## Abstract

This work presents the synthesis and application of magnetic Fe_3_O_4_ nanoparticles supported on baobab seed-derived biochar (Fe_3_O_4_/BSB) for removing Congo red (CR) dye from aqueous solutions through an oxidative process. The biochar support offered a porous structure with a surface area of 85.6 m^2^/g, facilitating uniform dispersion of Fe_3_O_4_ nanoparticles and efficient oxidative activity. Fourier-transform infrared (FT–IR) spectroscopy analysis confirmed surface fictionalization after Fe_3_O_4_ incorporation, while scanning electron microscopy (SEM) images revealed a rough, porous morphology with well-dispersed nanoparticles. Thermogravimetric analysis (TGA) demonstrated enhanced thermal stability, with Fe_3_O_4_/BSB retaining ~40% of its mass at 600 °C compared to ~15–20% for raw baobab seeds. Batch experiments indicated that operational factors such as pH, nanoparticles dosage, and initial dye concentration significantly affected removal efficiency. Optimal CR removal (94.2%) was achieved at pH 4, attributed to stronger electrostatic interactions, whereas efficiency declined from 94.1% to 82.8% as the initial dye concentration increased from 10 to 80 mg/L. Kinetic studies showed that the pseudo-second-order model accurately described the oxidative degradation process. Reusability tests confirmed good stability, with removal efficiency decreasing only from 92.6% to 80.7% after four consecutive cycles. Overall, Fe_3_O_4_/BSB proves to be a thermally stable, magnetically recoverable, and sustainable catalyst system for treating dye-contaminated wastewater.

## 1. Introduction

Global population growth, combined with rapid industrialization, has intensified anthropogenic pressures on the environment. Industrial effluents, particularly from sectors such as cosmetics, textiles, and plastics, contain substantial loads of synthetic organic and inorganic pollutants that contribute significantly to aquatic contamination. Elevated dye concentrations in water bodies not only reduce light penetration and impair photosynthetic activity but also yield biotransformation products with high toxicity to aquatic biota [1,2,3,4]. Effective removal of these contaminants prior to effluent discharge is, therefore, critical. Congo red (CR), a widely used anionic dye in multiple industrial processes, is of particular concern due to its persistence, ecological toxicity, and potential biological hazards.

Several techniques have been investigated for the elimination of dyes from wastewater, including adsorption, membrane separation, nanofiltration, electrochemical processes, and advanced oxidation processes (AOPs) [1,2]. Among these, AOPs are recognized as highly efficient due to their rapid oxidation rates, strong mineralization capacity, and lower likelihood of producing secondary contaminants. These processes employ potent oxidizing agents, such as hydrogen peroxide (H_2_O_2_), ozone (often combined with H_2_O_2_), sodium hypochlorite, potassium permanganate, and potassium ferrate (VI). Their efficiency primarily arises from the in situ generation of hydroxyl radicals (•OH), which are extremely reactive, non-selective species capable of degrading a broad spectrum of organic pollutants. The production of •OH radicals can occur via homogeneous reactions, like the classical Fenton reaction, or through heterogeneous catalytic systems involving activated carbon, graphite, metal oxides, or supported metals [5]. Owing to their high oxidative potential, hydroxyl radicals play a pivotal role in pollutant breakdown, positioning AOPs as one of the most promising methods for wastewater treatment. Numerous studies have demonstrated the successful removal of dyes using AOP-based strategies such as Fenton and photo-Fenton systems, photocatalytic degradation, ozonation, electrochemical oxidation, sonochemical treatment, and combined oxidation processes [3,6,7,8,9,10].

Biochar (BC), a biomass-derived functional material, has recently gained attention as a sustainable alternative to conventional catalysts due to its high surface activity, porosity, structural versatility, and cost-effectiveness. However, its application in wastewater treatment is constrained by challenges related to separation, regeneration, and potential secondary pollution, as BC particles released into aquatic environments may enhance pollutant migration and resuspension in sediments [11,12]. To address these limitations, the development of BC-based magnetic nanoparticles has emerged as a promising strategy. The incorporation of magnetic nanoparticles, particularly Fe_3_O_4_, can markedly improve the separation and recyclability of BC. Fe_3_O_4_ nanoparticles, which exhibit strong magnetic properties, can be effectively loaded onto BC via methods such as co-precipitation, hydrothermal synthesis, and microwave-assisted processes [11,13,14]. Owing to their facile recovery, reusability, and broad applicability, Fe_3_O_4_ magnetic nanoparticles (MNPs) have attracted significant research interest [13]. Recent studies have demonstrated that Fe_3_O_4_–BC composites are highly efficient in the removal of organic dyes from aqueous media while being readily separable using an external magnetic field. Various biomass precursors, including rice husk [11], sewage sludge and woodchips [15], corn straw [16], and pomelo peel [17], have been successfully employed to produce magnetic BC adsorbents for dye removal.

In this study, baobab seeds (Adansonia digitata) were chosen over other agricultural biomasses due to their unique structural and chemical properties, which make them particularly suitable for dye removal. Rich in lignocellulosic content, baobab seeds produce biochar with enhanced carbonization and surface functionality. Compared with conventional agricultural wastes, baobab seed biochar exhibits exceptionally high specific surface area, well-developed porosity, and deep voids, providing abundant active sites for adsorption [18]. Its multifunctional surface chemistry, containing hydroxyl, carbonyl, and aromatic groups, further promotes strong interactions with dye molecules and other contaminants [19]. These intrinsic features make baobab seed biochar an excellent support for magnetic nanoparticles, improving dispersion, stability, and reusability in wastewater treatment applications. The novelty of this work lies in the first-time synthesis of magnetic Fe_3_O_4_ nanoparticles supported on baobab seed-derived biochar for the efficient removal of Congo red dye from aqueous media. While previous studies have utilized agricultural wastes for biochar production, the high surface area, well-defined porosity, and rich functional groups of baobab seed biochar offer a superior platform for nanoparticles immobilization. This sustainable approach not only valorizes an underutilized biowaste but also enhances removal efficiency, magnetic recovery, and reusability—addressing key challenges in dye wastewater treatment. This study focused on synthesizing the Fe_3_O_4_/baobab seed biochar nanoparticles and characterizing their structural and morphological properties. This study also investigated the influence of operational parameters on CR degradation and examined the degradation kinetics to understand the adsorption and catalytic behavior of the material. Finally, the regeneration and reusability of the synthesized nanoparticles were assessed to evaluate their potential for sustainable wastewater treatment applications.

## 2. Results and Discussions

### 2.1. Characterization

#### 2.1.1. SEM Characterization

**Figure 1:**
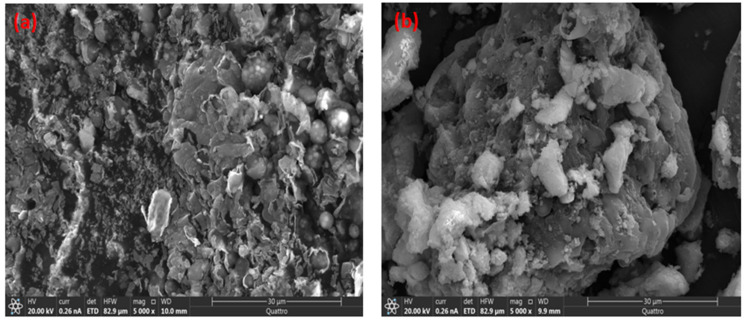
SEM micrographs of (**a**) BS and (**b**) Fe_3_O_4_/BSB nanoparticles.

The SEM images in Figure 1 reveal distinct differences in the morphology of raw baobab seeds (BS) and Fe_3_O_4_/BSB nanoparticles. In Figure 1a, the surface of BS appears relatively irregular and rough, with layered structures and fragmented particles, reflecting the inherent biochar texture after pyrolysis. The surface exhibits natural cavities and roughness, which are advantageous for the subsequent deposition of magnetic nanoparticles due to increased surface area and potential anchoring sites. In contrast, Figure 1b shows the Fe_3_O_4_/BSB nanoparticles, where the surface is noticeably rougher and covered with aggregated nanoparticles. The presence of spherical Fe_3_O_4_ particles on the biochar surface is evident, indicating successful deposition of magnetic nanoparticles onto the biochar matrix. This uniform distribution of Fe_3_O_4_ enhances the surface area and provides active sites for dye adsorption and catalytic degradation [20]. Additionally, the Fe_3_O_4_ nanoparticles appear well-anchored, suggesting strong interaction with the biochar support, which is expected to improve the stability and reusability of the composite in wastewater treatment applications.

#### 2.1.2. FTIR Characterization

**Figure 2:**
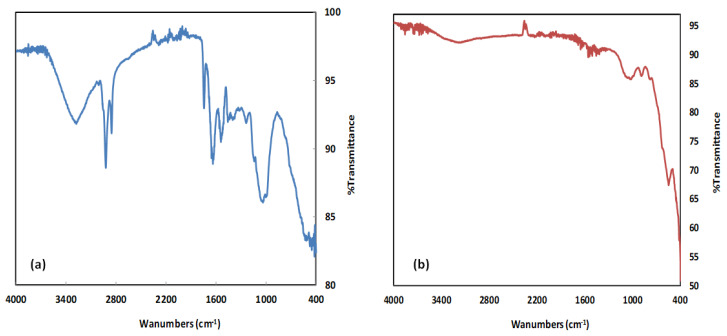
FTIR spectra of (**a**) BS, and (**b**) Fe_3_O_4_/BSB nanoparticles.

The FTIR spectra of BS and Fe_3_O_4_/BSB nanoparticles are shown in Table 1 and Figure 2. The spectrum of BS displayed a wide range of functional groups, many of which exhibited reduced intensity or disappeared altogether after nanoparticle formation. A broad absorption band at approximately 3259 cm^−1^ in the BS spectrum was attributed to O–H stretching vibrations of hydroxyl groups, including hydrogen-bonded hydroxyls, indicating their predominance in the raw material [21]. Additional peaks were observed at 2922 cm^−1^, 2032 cm^−1^, 1743 cm^−1^, 1541 cm^−1^, and 1056 cm^−1^, corresponding to C–H stretching of hemicellulose, C≡C stretching of alkynes [22], C=O stretching in lactones, ketones, and carboxylic anhydrides, C=C aromatic vibrations from lignin, and C–O stretching, respectively (Figure 2a) [21,22,23]. In contrast, the FTIR spectrum of Fe_3_O_4_/BSB nanoparticles (Figure 2b) showed a noticeable reduction in peak intensities, particularly due to the decomposition of carboxyl functional groups within the 4000–2000 cm^−1^ region. A distinct band at 1537 cm^−1^ suggested the retention of stable aromatic or graphitic structures [22]. As reported in earlier studies, metal–oxygen stretching vibrations typically occur in the range of 500–600 cm^−1^ [23], and a characteristic Fe–O absorption band was detected at 551 cm^−1^, confirming the successful incorporation of Fe_3_O_4_. Moreover, the peaks originally observed at 1056 and 1710 cm^−1^, assigned to C–O and C=O stretching, were either significantly weakened or absent in Fe_3_O_4_/BS compared to BS. This disappearance indicates that hemicelluloses, lignin, and cellulose components of the raw biomass underwent extensive depolymerization and degradation during the pyrolysis and nanoparticle synthesis process [23].

#### 2.1.3. TGA Analysis

The thermogravimetric analysis (TGA) profiles of BS and Fe_3_O_4_/BSB nanoparticles are presented in Figure S1. Both samples exhibit an initial weight loss below 150 °C, which is mainly attributed to the evaporation of physically adsorbed moisture and volatile compounds. In the temperature range of 200–450 °C, BS undergoes a sharp weight loss associated with the decomposition of major organic constituents, such as cellulose, hemicellulose, and lignin [22]. In contrast, the Fe_3_O_4_/BSB NPs show a more gradual mass reduction in this region, reflecting the improved thermal stability of the composite. Beyond 450 °C, BS continues to degrade extensively, leaving a small residual mass (15–20% at 600 °C). On the other hand, Fe_3_O_4_/BSB NPs retain a significantly higher residue (40% at 600 °C), which is attributed to the presence of thermally stable Fe_2_O_3_. These results indicate that the incorporation of Fe_3_O_4_ nanoparticles enhances the structural integrity of the biomass matrix and effectively delays thermal degradation, confirming successful interaction between the inorganic phase and the biomass support. Comparable findings have been reported for other biomass–metal oxide systems. Similarly, Fe_3_O_4_-loaded polymer composites such as LDPE/hematite blends exhibited a shift in decomposition onset temperature by ~20 °C relative to the pure matrix, attributed to the barrier effect of iron oxide nanoparticles restricting chain scission [24].

#### 2.1.4. BET Analysis

**Figure 3:**
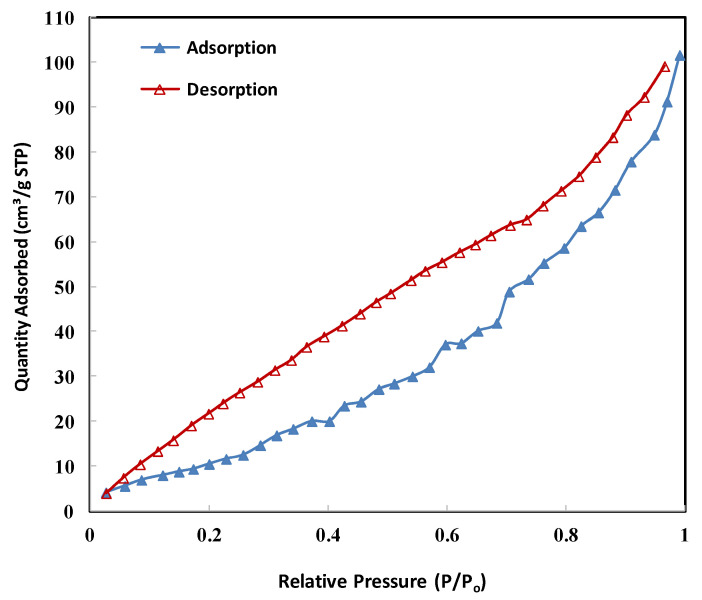
The Fe_3_O_4_/BSB NPs N_2_ adsorption/desorption isotherms.

The nitrogen adsorption–desorption isotherm of the Fe_3_O_4_/biochar-supported baobab seed (Fe_3_O_4_/BSB) nanoparticles is presented in Figure 3. The isotherm profile provides valuable insights into the textural and porous characteristics of the synthesized material. The adsorption curve initially shows a sharp rise at low relative pressures (*p*/*p*_0_ < 0.05), which can be attributed to monolayer–multilayer adsorption on the surface of the nanoparticles. This indicates the presence of micropores and surface adsorption sites. As the relative pressure increases, the adsorption volume continues to rise gradually, followed by a more pronounced increase at higher *p*/*p*_0_ values (>0.3), suggesting the coexistence of mesopores within the structure. The desorption branch does not fully overlap with the adsorption curve, leading to a visible hysteresis loop, which is characteristic of mesoporous materials. Such hysteresis behavior typically reflects capillary condensation within mesopores and confirms the heterogeneous pore structure of the Fe_3_O_4_/BSB nanoparticles. The BET analysis in the relative pressure range of 0.05–0.30 revealed a surface area of 85.6 m^2^/g, with a total pore volume of 0.173 cm^3^/g and an average pore diameter of 12.6 nm. According to IUPAC classification, these results confirm that the material is predominantly mesoporous, while also containing microporous regions that contribute to the surface area [25]. The relatively high surface area and hierarchical pore structure of Fe_3_O_4_/BSB nanoparticles are advantageous for adsorption and catalytic applications, as they provide abundant active sites and facilitate the diffusion of adsorbates. The mesoporosity is especially important for accommodating larger dye molecules, such as Congo red, enhancing both adsorption and oxidative degradation efficiency. Similar isotherm behavior has been reported for biochar-supported magnetic nanoparticles, highlighting the role of biochar in creating a porous matrix, while Fe_3_O_4_ nanoparticles further enrich the surface functionality and catalytic activity [26].

### 2.2. Degradation Studies

#### 2.2.1. Effect of Contact Time

**Figure 4:**
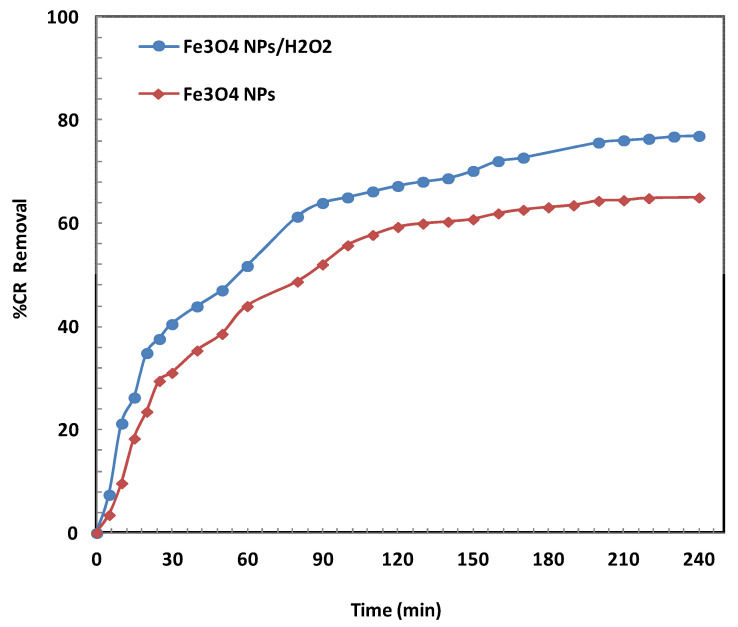
Effect of addition of H_2_O_2_ on the decomposition of CR under the conditions of 30 mL of 30 mg/L CR, 1 mL of H_2_O_2_, 0.1 g of Fe_3_O_4_/BSB NPs, 25 °C, 120 rpm for 4 h.

To assess the role of H_2_O_2_ in the decomposition system, two comparative experiments were carried out using bare Fe_3_O_4_/BSB NPs, with and without H_2_O_2_. As illustrated in Figure 4, the addition of H_2_O_2_ markedly improved CR degradation, enhancing removal efficiency by approximately 15% compared to the system without H_2_O_2_. After 4 h, CR removal reached 77% in the presence of H_2_O_2_ versus 65% in its absence. This improvement is attributed to the generation of hydroxyl radicals (•OH) through the Fenton-like reaction between Fe^2+^ ions on the nanoparticle surface and H_2_O_2_, which accelerates the oxidative breakdown of CR molecules. Based on these findings, all subsequent experiments were performed in the presence of H_2_O_2_ [27].

#### 2.2.2. Influence of pH

**Figure 5:**
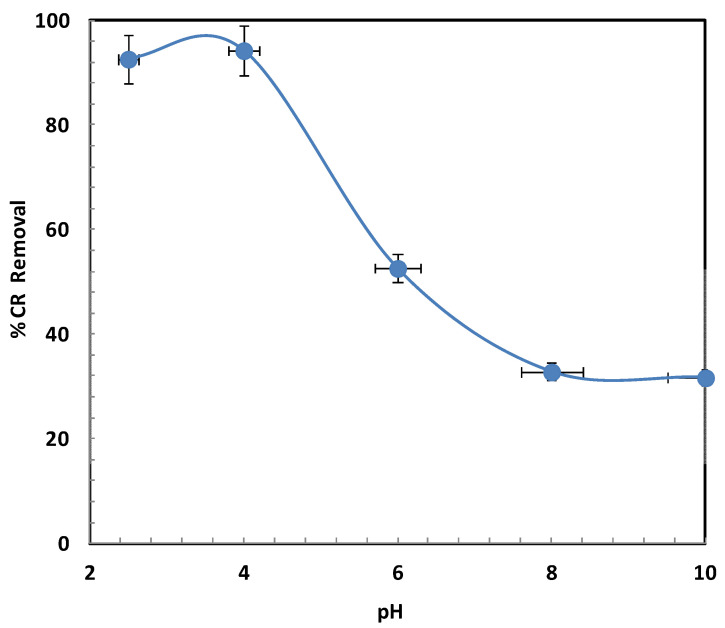
Effect of pH on CR degradation.

The pH of the CR solution is a key factor influencing its removal, as it directly affects the surface charge characteristics of the Fe_3_O_4_/BSB NPs as well as the ionization degree of the CR dye molecules [28]. In this study, the effect of pH (2.0–10.0) was examined under fixed conditions: CR concentration of 30 mg/L, contact time of 24 h, and Fe_3_O_4_/BSB NPs dosage of 0.1 g. As shown in Figure 5, the maximum removal efficiency (94.2%) was achieved at pH 4. This high efficiency at acidic pH can be attributed to protonation of the Fe_3_O_4_/BSB NPs surface functional groups, such as hydroxyl and carboxyl moieties, leading to an overall positive surface charge. The positively charged surface strongly attracts the negatively charged sulfonate groups of CR molecules via electrostatic attraction, promoting rapid adsorption. However, increasing the solution pH from 4 to 10 resulted in a gradual decrease in removal efficiency, dropping sharply to 31.7% at pH values above 8. This decline is primarily due to deprotonation of the adsorbent surface functional groups, which imparts a negative surface charge to the Fe_3_O_4_/BSB NPs [29]. At alkaline pH, both the adsorbent and CR molecules bear negative charges, resulting in significant electrostatic repulsion that hinders CR molecules from approaching and binding to the adsorbent surface. In addition, higher OH^−^ concentrations in alkaline solutions can compete with CR anions for available active sites, further reducing adsorption capacity. Similar pH-dependent adsorption behavior for CR has been widely reported in the literature [30,31,32], reinforcing the importance of surface charge control in optimizing dye removal. From a practical perspective, this suggests that pretreatment steps involving pH adjustment could significantly enhance CR removal efficiency in wastewater treatment applications.

#### 2.2.3. Influence of Adsorbent Dose

**Figure 6:**
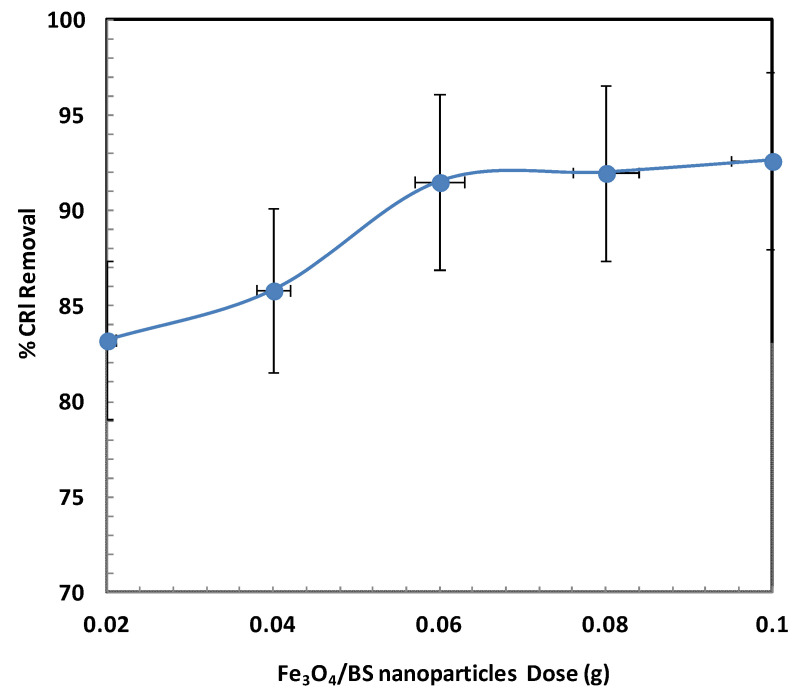
Effect of Fe_3_O_4_/BSB NPs dose.

Figure 6 presents the oxidative degradation efficiency of CR at varying Fe_3_O_4_/biochar nanoparticle dosages, ranging from 0.02 g to 0.1 g. The data clearly indicate that hydroxyl radicals generated in the presence of Fe_3_O_4_/BSB NPs play a crucial role in breaking down the CR dye molecules. As the Fe_3_O_4_/BSB NPs dosage increased from 0.02 g to 0.06 g, the degradation efficiency rose notably from 83% to 93%, which can be attributed to the increased number of active sites available for radical generation and dye adsorption. These active sites facilitate enhanced interaction between the catalyst and dye molecules, leading to more efficient oxidative breakdown. However, further increasing the dosage from 0.06 g to 0.1 g did not result in any significant improvement in degradation efficiency, indicating a plateau effect. This saturation could be due to particle agglomeration at higher dosages, which reduces the available surface area and limits mass transfer between the dye molecules and catalytic sites [32]. Similar trends have been reported in other heterogeneous catalytic degradation studies, where optimal catalyst dosage is critical for maximizing efficiency without introducing diffusion limitations or radical scavenging effects [27,32].

#### 2.2.4. Influence of Initial Concentration

**Figure 7:**
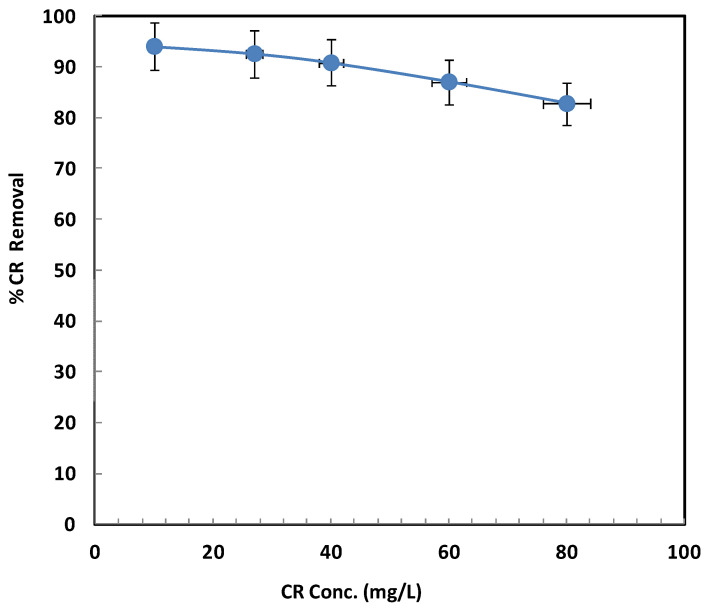
Effect of initial CR concentration.

Figure 7 presents the influence of varying initial CR concentrations, ranging from 10 to 80 mg/L, on removal efficiency under fixed conditions: pH 3, Fe_3_O_4_/BSB NPs dosage of 0.1 g, and a temperature of 25 °C. The results clearly indicate a negative correlation between initial CR concentration and removal efficiency. At a low concentration of 10 mg/L, the removal efficiency reached 94.1%. However, as the CR concentration increased to 80 mg/L, the efficiency dropped to 82.8%. This decline can be explained by the finite number of active sites available on the Fe_3_O_4_/BSB nanoparticle surfaces. Since the catalyst dosage remained constant, an increase in CR concentration meant that more dye molecules were competing for the same limited active sites. At higher concentrations, the available sites become rapidly saturated, leaving excess CR molecules in solution without sufficient interaction with the adsorbent [33]. Furthermore, higher dye concentrations can lead to increased solution viscosity and hinder mass transfer, slowing the diffusion of CR molecules to the catalyst surface. Additionally, in advanced oxidation processes, high pollutant concentrations can act as radical scavengers, reducing the number of hydroxyl radicals available for oxidative degradation [33,34,35]. These combined effects not only reduce removal efficiency but may also prolong the reaction time required for complete degradation. Similar trends have been observed in previous studies, such as those by Jain et al. [36] and Shaban et al. [37], highlighting that beyond a certain threshold, increasing dye concentration results in diminished adsorption and degradation performance due to both surface saturation and kinetic limitations.

#### 2.2.5. Adsorption Kinetics

**Figure 8:**
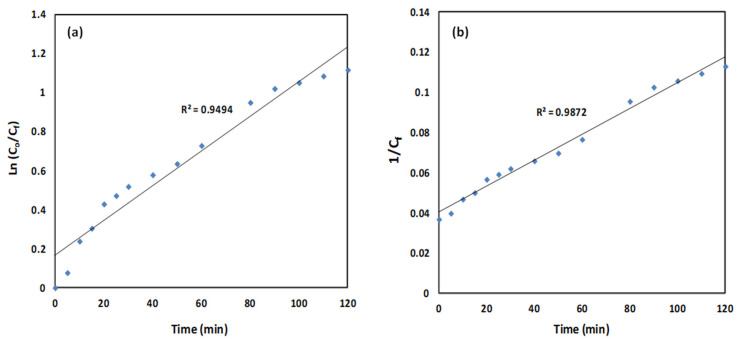
(**a**) ln(*C*_0_*/C_f_*) and (**b**) 1/*C_f_* over time (*C*_0_ = 30 mg/L, Fe_3_O_4_/BSB NPs = 0.1 g, pH 4).

In this study, the kinetics of the oxidation process were analyzed using pseudo-first-order and pseudo-second-order models (Equations (1) and (2)). The pseudo-first-order model is appropriate when the concentration of reactive species, such as oxidants, is in large excess and remains essentially constant, so that the reaction rate depends primarily on the pollutant concentration. The pseudo-second-order model is applied when the reaction rate is influenced by both the pollutant and interactions with the nanoparticle surface, as commonly observed in heterogeneous catalytic systems. Together, these models provide an effective empirical description of the degradation kinetics, allowing for the estimation of rate constants and enabling comparison of nanoparticle performance.

(1) $$ln\frac{C_{0}}{C_{f}}=k_{1}t\;$$

(2) $$\frac{1}{C_{f}}=k_{2}t+\frac{1}{C_{0}}$$

where *C*_0_ and *C_f_* are the initial and final concentrations of CR; *t* is time (min), and *k*_1_ and *k*_2_ are the rate constants. The calculated rate constants and correlation coefficients (*R*^2^) are presented in Table 2 and Figure 8. Both models fit the experimental data well, but the pseudo-second-order model shows a superior correlation (*R*^2^ = 0.9872), indicating that chemisorption predominates in the removal process. These kinetic results are consistent with the observed concentration-dependent behavior: at higher dye concentrations, the limited availability of active sites slows adsorption, as reflected in both the reduction in removal efficiency and the pseudo-second-order kinetic model. Comparative studies on Fe_3_O_4_-based nanoparticles for CR removal also report pseudo-second-order kinetics, emphasizing the role of surface interactions and chemical adsorption mechanisms. Collectively, these findings suggest that optimizing nanoparticle dosage relative to dye concentration is essential for maintaining high removal efficiency and achieving effective wastewater treatment [35].

#### 2.2.6. Degradation Mechanism of CR Dye

The degradation mechanism of Congo red (CR) dye using Fe_3_O_4_/BSB nanoparticles involves a synergistic process of adsorption followed by oxidative degradation. Initially, the porous biochar matrix, with its abundance of functional groups, facilitates the adsorption of CR molecules through electrostatic attraction, π–π interactions, and hydrogen bonding. Once immobilized on the Fe_3_O_4_/BSB surface, the Fe_3_O_4_ nanoparticles play a catalytic role by activating oxidants (such as H_2_O_2_), generating reactive oxygen species (ROS), including hydroxyl radicals (•OH) and superoxide anions (O_2_•^−^). These highly reactive species attack the azo bonds (-N=N-) and aromatic rings of CR, leading to bond cleavage, decolorization, and subsequent breakdown into smaller intermediates. Continued oxidation mineralizes these intermediates into low-toxicity end products such as CO_2_, H_2_O, and inorganic ions (e.g., SO_4_^2−^, NO_3_^−^). The magnetic nature of the composite allows for easy recovery after treatment, while the stability of the biochar support enhances reusability. This combined adsorption–oxidation pathway ensures efficient and sustainable degradation of CR dye.

#### 2.2.7. Recycling Test

**Figure 9:**
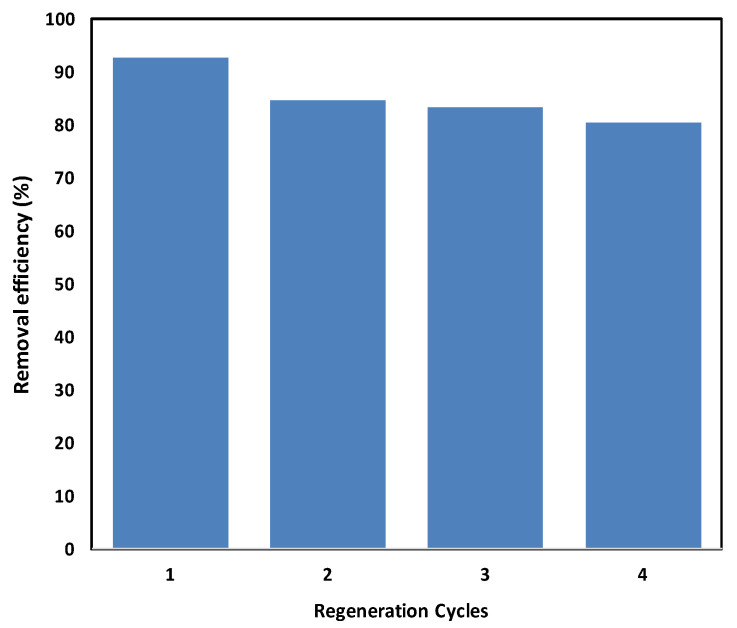
Recycling of Fe_3_O_4_/BSB nanoparticles for multiple adsorption-desorption of CR.

An important parameter in evaluating the practical applicability of an adsorbent is its reusability. The regeneration performance of Fe_3_O_4_/BSB nanoparticles for CR removal is presented in Figure 9 over four consecutive cycles. After the first cycle, the nanoparticles achieved a removal efficiency of 92.6%. Although this efficiency gradually decreased with repeated use, it remained relatively high at 80.7% after the fourth cycle (Figure 9), indicating only a modest decline. The slight reduction in regeneration efficiency can be attributed to possible alterations in the surface structure of the nanoparticles and partial loss of active sites or mineral components during repeated adsorption–desorption processes [38]. Nevertheless, the results demonstrate that Fe_3_O_4_/BSB NPs maintain considerable structural integrity and adsorption capacity, highlighting their stability and suitability for multiple reuse cycles. Moreover, the ability to retain high removal efficiency over multiple cycles has significant practical implications, as it reduces operational costs and enhances sustainability in wastewater treatment applications. The observed reusability is comparable with other Fe_3_O_4_-based nanoparticles reported in recent studies [39,40], confirming that magnetic biochar nanoparticles offer a promising, cost-effective, and environmentally friendly solution for the removal of synthetic dyes from aqueous systems.

## 3. Materials and Methods

### 3.1. Chemical

Sodium hydroxide (NaOH), hydrochloric acid (HCl), sulfuric acid (H_2_SO_4_), ammonium hydroxide (NH_4_OH), ferrous sulfate heptahydrate (FeSO_4_·7H_2_O), and ferric chloride hexahydrate (FeCl_3_·6H_2_O) were employed in the preparation of magnetic nanoparticles. NaOH and HCl were primarily used to adjust and maintain the pH of the reaction solutions. Congo red (CR, 98% purity) served as the model dye for degradation studies. All reagents were of analytical grade and obtained from Merck (Rahway, NJ, USA).

### 3.2. Synthesis of Fe_3_O_4_/Biochar Nanoparticles

**Figure 10:**
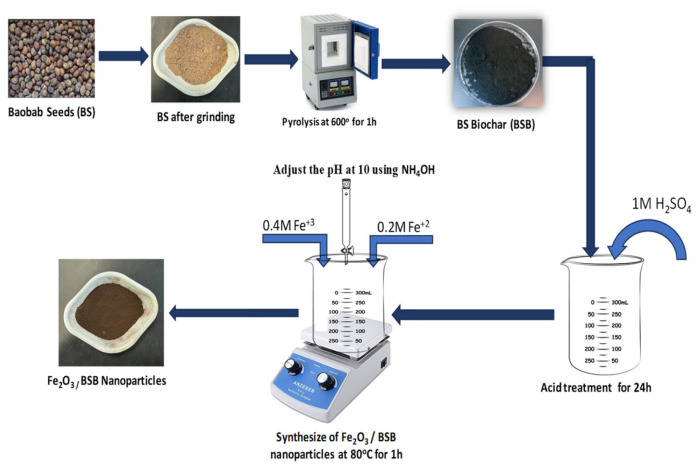
Synthesize Fe_3_O_4_/BSB nanoparticles.

Baobab seeds (BS) were obtained from a local market, washed with distilled water, and oven-dried at 80 °C for 24 h. The dried seeds were milled into powder, and particles sized between 125 and 250 µm were collected for further experiments. Biochar was generated by pyrolyzing the seed powder in a furnace under oxygen-limited conditions at 600 °C with a heating rate of 10 °C/min and a retention time of 2 h, resulting in baobab seed biochar (BSB). The obtained BSB was then activated by soaking in 1 M H_2_SO_4_ at ambient temperature with continuous stirring for 24 h, followed by repeated washing with distilled water until the pH reached neutrality. The Fe_3_O_4_/BSB nanoparticles were prepared through a modified chemical co-precipitation method [20]. Specifically, 5.56 g of FeSO_4_·7H_2_O and 6.49 g of FeCl_3_·6H_2_O were dissolved in 100 mL of distilled water preheated to 80 °C under constant stirring. Subsequently, 10 mL of 25% NH_4_OH was slowly added to adjust the pH to ~10. Afterward, 10 g of acid-treated BSB was introduced into the solution and stirred for 1 h, allowing for uniform anchoring of Fe_3_O_4_ nanoparticles on the biochar matrix. The material was vacuum-filtered, washed with distilled water to neutrality, dried at 60 °C, and calcined at 200 °C for 4 h to enhance stability and surface properties. Figure 10 shows the steps of the Fe_3_O_4_/BSB nanoparticles synthesis.

### 3.3. Instrument Analysis

Fourier-transform infrared (FT–IR) spectroscopy (Cary 630) in the 4000–400 cm^−1^ range was used to identify surface functional groups. Morphological features were examined via scanning electron microscopy (SEM, FEI QUANTA FEG 250, FEI Company, Hillsboro, OR, USA) at 15 keV. The surface area of Fe_3_O_4_/BSB nanocomposite was measured by nitrogen adsorption at −196 °C using Brunauer–Emmett–Teller (BET) (ASAP 2020, Micromeritics Instrument Corp., Norcross, GA, USA) after degassing at 200 °C for 4 h, with the BET method applied for calculation. Thermal stability and weight loss were assessed through (TG–DTA, STA 6000, PerkinElmer Inc., Waltham, MA, USA) from 30–800 °C at 10°C/min under N_2_ flow (20 mL/min). Biochar yield, calculated via Equation (3), was 25.84%, reflecting the conversion efficiency of baobab seeds.

(3) $$Biochar\;\;yield\;\left(\%\right)=\frac{mass\;of\;biochar}{mass\;of\;dried\;smaple}\times 100\;\;\;\;\;\;$$

### 3.4. Experimental Procedures

Congo red (CR) degradation was carried out by treating 20 mL of a 20 mg/L dye solution with 0.1 g of Fe_3_O_4_/BSB nanoparticles and 0.05 M H_2_O_2_ at ambient conditions (pH and 25 °C). The mixture was stirred at 100 rpm for 24 h to ensure effective contact between the nanocomposite, oxidant, and dye. After filtration, residual dye concentration was measured at 557 nm using a UV–visible spectrophotometer (UV-1700, Shimadzu, Corporation, Kyoto, Japan). Batch experiments were performed to study the effects of nanocomposite dosage (0.05–0.15 g), H_2_O_2_ concentration (0–0.1 M), and solution pH (2–10, adjusted with 1 M HCl or NaOH). Kinetic studies were conducted at pH 3, 25 °C, 0.1 g nanocomposite, and 27 mg/L dye, stirring at 120 rpm, with samples collected at predetermined intervals (0–120 min).

The reusability of the Fe_3_O_4_/BSB nanoparticles was evaluated over four consecutive cycles. After each degradation experiment, the dye-laden nanoparticles were washed using 0.1 M NaOH, followed by sequential washing with distilled water and 0.1 M HCl until the pH stabilized at approximately 7.0. The regenerated material was then reused in a fresh dye solution under identical oxidation conditions. The removal efficiency in each cycle was measured to assess the stability and practical applicability of the nanoparticles. Dye removal efficiency was calculated via Equation (4), providing quantitative insight into the catalytic degradation process. All experiments were conducted in duplicate.

(4) $$Removal\;efficiency\;\left(\%\right)=\frac{\left(C_{0}-C_{f}\right)}{C_{0}}\times 100$$

where *C*_0_ represents the initial CR concentration (mg/L) and *C_f_* represents the final CR concentration (mg/L).

## 4. Conclusions

This study demonstrated that Fe_3_O_4_/BSB nanoparticles are highly effective for removing Congo red from aqueous solutions, with performance strongly dependent on solution pH, adsorbent dosage, and initial dye concentration. Maximum removal (94.2%) was observed at acidic pH 4, where protonation of surface functional groups enhances electrostatic attraction between the positively charged nanoparticles and anionic CR molecules. At higher pH, deprotonation leads to electrostatic repulsion and competition with OH^−^ ions, reducing adsorption efficiency. The oxidative degradation of CR increased with Fe_3_O_4_/BSB dosage, peaking at 0.06 g; further increases offered no improvement, likely due to particle agglomeration, limited mass transfer, and reduced light penetration in photo-assisted systems. Higher initial dye concentrations decreased efficiency from 94.1% (10 mg/L) to 82.8% (80 mg/L) due to active-site saturation, hindered diffusion, and potential radical scavenging. Kinetic studies indicated a pseudo-second-order model, suggesting chemisorption predominates. Reusability tests confirmed the nanoparticles’ practical applicability, maintaining 81% removal after four cycles, demonstrating structural stability and multi-cycle potential. Overall, Fe_3_O_4_/BSB nanoparticles provide a cost-effective, stable, and sustainable solution for CR removal, with potential for industrial-scale wastewater treatment of dye-contaminated water.

## Figures and Tables

**Table 1:** FTIR functional groups on BS and Fe_3_O_4_/BSB nanoparticles.

Functional Groups	Wavenumber (cm^−1^)		Reference
	Fe_3_O_4_/BSB NPs	BS	
O–H stretching vibrations of alcohols and carboxylic groups.	3128.53	3259	3600–3400
C–H stretching vibrations of alkanes.	-	2852.72, 2922.16	3000–2800
C≡C stretching vibrations of alkynes.	2349.34	2032.97	2260–2100
C=O stretching vibrations (lactones, ketones, anhydrides).	-	1743.65	1740–1730
C=C stretching vibrations of alkenes.	1653.70	1637.56	1650–1600
C=C aromatic ring vibrations (lignin).	1537.26	1541.12	1600–1500
C–O stretching vibrations of ether/ester.	1026.17	1056.99, 1238.30	1300–1000
C=C–H bending vibrations.	871.82	-	1000–675
Fe–O vibrations.	551.64	-	600–500

**Table 2:** Kinetic models, constant parameters, and correlation coefficients.

Initial CR Con. (mg/L)	Pseudo First-Order Model		Pseudo Second-Order Model	
	*k*_1_ (min^−1^)	*R* ^2^	*k*_2_ (L/mg min)	*R* ^2^
30	0.0089	0.9494	0.0006	0.9872

## Data Availability

Data will be made available upon request.

## Supplementary Materials

The following supporting information can be downloaded at: https://www.mdpi.com/article/10.3390/ijms26178499/s1.
