# Supplementary figures and images for: Biomass-Derived Magnetic Fe3O4/Biochar Nanoparticles from Baobab Seeds for Sustainable Wastewater Dye Remediation
Source: Int J Mol Sci. 2025 Sep 1;26(17):8499. doi: 10.3390/ijms26178499 (PMC12429094; doi:10.3390/ijms26178499)

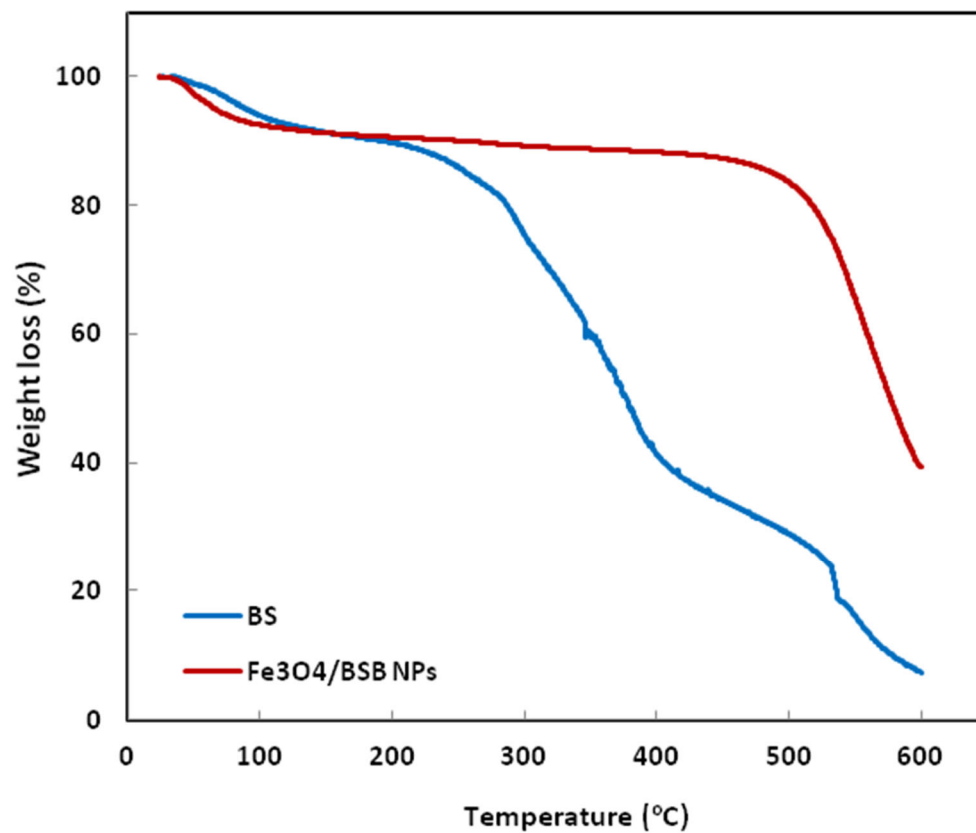

**Figure S1.** TGA curves of BS and Fe<sub>3</sub>O<sub>4</sub>/BSB NPs .

Supplement: Supplementary file 1 [file ijms-26-08499-s001.zip › ijms-3858070-supplementary.pdf]
